# Modification of Collagen Properties with Ferulic Acid

**DOI:** 10.3390/ma13153419

**Published:** 2020-08-03

**Authors:** Beata Kaczmarek, Katarzyna Lewandowska, Alina Sionkowska

**Affiliations:** Department of Biomaterials and Cosmetics Chemistry, Faculty of Chemistry, Nicolaus Copernicus University in Torun, Gagarin 7, 87-100 Toruń, Poland; beata.kaczmarek@umk.pl (B.K.); reol@umk.pl (K.L.)

**Keywords:** collagen, ferulic acid, rheology, thin films

## Abstract

Collagen materials are widely used in biomedicine and in cosmetics. However, their properties require improvement for several reasons. In this work, collagen solution as well as collagen films were modified by the addition of ferulic acid (FA). Thin collagen films containing FA were obtained by solvent evaporation. The properties of collagen solution have been studied by steady shear tests. The structure and surface properties of collagen thin films were studied. It was found that for collagen solution with 5% addition of FA, the apparent viscosity was the highest, whereas the collagen solutions with other additions of FA (1%, 2%, and 10%), no significant difference in the apparent viscosity was observed. Thin films prepared from collagen with 1 and 2% FA addition were homogeneous, whereas films with 5% and 10% FA showed irregularity in the surface properties. Mechanical properties, such as maximum tensile strength and elongation at break, were significantly higher for films with 10% FA than for films with smaller amount of FA. Young modulus was similar for films with 1% and 10% FA addition, but bigger than for 2% and 5% of FA in collagen films. The cross-linking of collagen with ferulic acid meant that prepared thin films were elastic with better mechanical properties than collagen films.

## 1. Introduction

Collagen is widely used for the preparation of several materials for tissue engineering applications [1,2,3,4,5,6,7,8,9,10,11,12,13,14,15,16]. It can be used in several forms, i.e., in the form of a gel, thin films, and a 3D sponge [1,2]. A very important group of materials for biomedical applications is collagen composites with inorganic particles and/or with another polymer [3,4,5,6,7,8,9,10,11,12,13,14,15,16]. Although every year several new papers are published regarding collagen composites, still there is a great need for the progress of composite biomaterials, which are effective for tissue engineering applications. Due to low durability to external factors, collagen requires stabilization. There are physical and chemical methods of improvement of collagenous structure. It is commonly known that this process requires the chemical agent or the physical phenomenon that induces stable intra- or intermolecular chemical bonds formation.

In this study, we use ferulic acid (FA) to modify collagen materials for potential biomedical and cosmetic applications. Biomedical applications of modified collagen may include wound dressing, artificial skin, bone tissue engineering, and many others. Ferulic acid is natural polyphenolic acid, which may be extracted from plant cell walls. It is well known as a component of herbal extracts with activity against different diseases due to its antioxidant properties [17]. Antioxidant activity has also been used for preparation of new materials for biomedical applications, because ferulic acid also has low toxicity and possesses many physiological functions such as anti-inflammatory, antimicrobial activity, anticancer, and antidiabetic effects [18,19]. It has been shown that some phenolic antioxidants such as ferulic acid prevented oxidation of collagen in vitro, and we can expect that ferulic acid may prevent tissue injury by oxidants, including peroxynitrite in vivo [20].

It was found that ferulic acid inhibits collagen self-association [21]. The inhibitory efficiency depends on the concentration of ferulic acid and temperature. Spectroscopic measurements indicated that collagen retains its unique triple helical structure in the presence of ferulic acid. Enzymatic studies suggested that FA protects collagen from enzymatic degradation. The inhibition of collagen fibrillation during its accumulation can be a therapeutic way to limit the fibrosis.

There are several papers that have been already published regarding collagen materials with ferulic acid incorporated in the materials. New composite electrospun nanofibres based on polycaprolactone and collagen hydrolysate loaded with ferulic acid were proposed by Kumar et al. [22]. The proposed material has been studied for the potential treatment of chronic wounds. Next, the thermosensitive FA-gelatin/chitosan/glycerol phosphate (FA-G/C/GP) hydrogel was developed by Cheng et al. [23]. The proposed hydrogel was applied as a sustained release system of FA to treat nucleus pulposus (NP) cells from the damage caused by oxidative stress. It has been concluded that the above-mentioned hydrogel may be applied in minimally invasive surgery for nucleus pulposus regeneration.

The possible therapeutic effects of FA-incorporated chitosan/gelatin/glycerol phosphate hydrogel on hydrogen-peroxide-induced oxidative stress NP cells was studied. The results showed that the release of FA from the hydrogel could decrease H_2_O_2_-induced oxidative stress [24]. FA-incorporated chitosan/gelatin/glycerol phosphate hydrogel could be used to treat the degenerative disc in the early stage before it developed into the latter irreversible stages.

The aim of this work was to study the influence of ferulic acid on rheological properties of collagen solution and the morphology and mechanical properties of thin films obtained from collagen/ferulic acid complex. 

## 2. Materials and Methods

### 2.1. Materials 

Collagen was isolated from rat tail tendons, which are the biological wastes found using the method published by us previously [25]. Collagen was dissolved in 0.1 M acetic acid (PolAura, Zabrze, Poland) at 1% concentration. Ferulic acid (Linegal, Warsaw, Poland) was dissolved and heated in 0.1 M acetic acid at 1% concentration as well. Then, it was added to collagen solution as 1%, 2%, 5%, and 10% addition based on collagen content. Collagen/ferulic acid mixture was mixed for 1h on magnetic stirred (IKA, Warsaw, Poland). Such complex solutions were then used for the study of rheological properties. Prepared solutions were also placed on the plastic holders, and thin films were obtained by the solvent evaporation. 

### 2.2. Fourier Transform Infrared Spectroscopy—Attenuated Total Reflectance (FTIR–ATR) 

FTIR-ATR spectra were made for each type of collagen/FA film in the range 1800–800 cm^−1^ by the spectrometer (Nicolet iS110, ThermoFisher Scientific, Waltham, MA, USA) equipped by diamond crystal with the resolution 4 cm^−1^. Spectra were taken with 64 scans. 

### 2.3. Scanning Electron Microscope (SEM)

The morphology of the obtained collagen/ferulic acid films were studied using Scanning Electron Microscope (SEM; LEO Electron Microscopy Ltd., Cambridge, UK). Samples were covered by gold to form the conductive surface for the electron beam interaction. SEM images were made with resolution 200 µm.

### 2.4. Mechanical Properties

Young Modulus (E_mod_), maximum tensile strength (σ_max_), and elongation at break (dl) were measured with the use of testing machine Z.05 (Zwick/Roell, Ulm, Germany) with the initial force 0.1 MPa and the velocity of 5 mm/min. Measurement was carried out in room conditions (n = 5). 

### 2.5. Steady Shear Tests

The steady shear flow properties were measured at a shear rate of 20 to 1230 s^−1^ at different temperatures (20, 25, 30, and 35 °C) using a rotational viscometer, Bohlin Visco 88 (Malvern Panalytical, Malvern, UK) with concentric cylinder, equipped with a heating system. The Ostwald de Waele model (Equation (1)) was used to fit the experimental data, which is suitable for describing the relationship between shear stress and shear rate in the collagen solution [26,27,28]

(1) $$\tau =k{\dot{\gamma }}^{n}$$

where *τ* is shear stress (Pa), $\dot{\gamma }$ is shear rate (1/s), and *n* and *k* are rheological parameters known as non-Newtonian index (dimensionless) and consistency index (Pa s^n^), respectively.

The viscosity dependence of the temperature was used to calculate the activation energy of viscous flow of the collagen solutions with different additions of FA applying the Arrhenius equation (Equation (2)) [29,30]

(2) $$\eta_{a}=Aexp\left(\frac{E_{a}}{RT}\right)$$

where *E_a_* is the activation energy of viscous flow (kJ mol^−1^), R is the gas constant (8.314 kJ mol^−1^ K^−1^), and T is the absolute temperature (K). The activation energy of viscous flow (*E_a_*) is determined from the slope of ln *η_a_* vs. 1/T curve.

## 3. Results

### 3.1. Fourier Transform Infrared Spectroscopy—Attenuated Total Reflectance (FTIR–ATR) 

**Figure 1:**
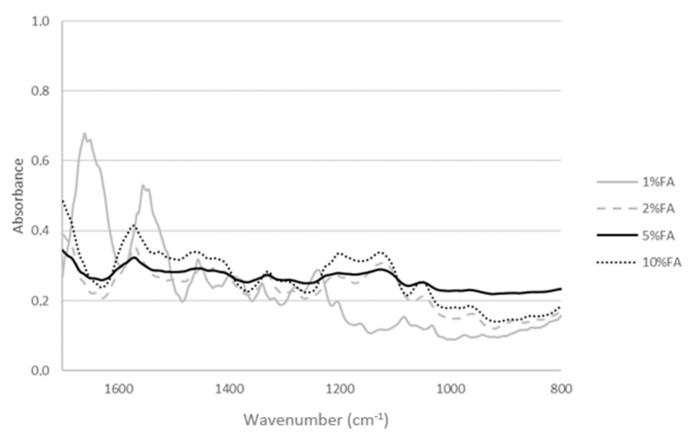
The Attenuated Total Reflectance (ATR) spectra of collagen thin films with ferulic acid addition.

**Figure 2:**
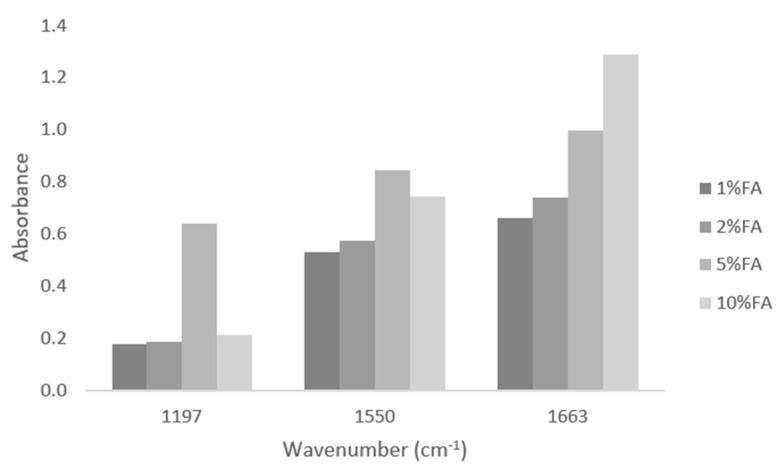
The asborbance values for the peaks at 1197, 1150, and 1663 cm^−1^.

**Figure 3:**
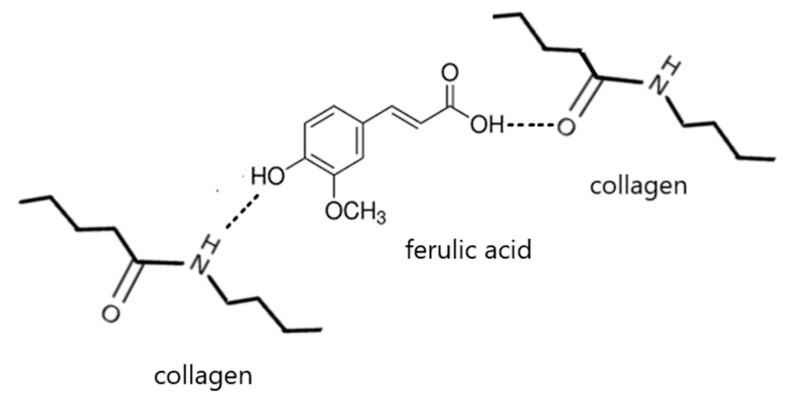
The schematic cross-linking process between collagen molecules and ferulic acid.

The FTIR-ATR spectra allow one to observe changes in the structure of collagen-ferulic acid complexes, which depend on the FA content (Figure 1 and Figure 2). The 1% addition of ferulic acid results in the high peak in 1663 cm^−1^ characteristic for amide I band attributed to the stretching vibrations of peptide C=O groups. Higher ferulic acid content results in the decrease of amide I band as a result of strong hydrogen bonds formed between collagen and ferulic acid (Figure 3). Similarly, 1550 cm^−1^ characteristic peak from amide II decreases with increasing amount of FA. This suggests that hydrogen bonds are formed between N–H group, as this peak is attributed to the N–H bending vibrations. The peak from the O-H group in 1197 cm^−1^ increases with increasing ferulic acid amount, as it remains uncross-linked by collagen. It may be assumed that the important changes are observed in secondary structure of collagen as a result of ferulic acid addition. 

### 3.2. Scanning Electron Microscopy (SEM)

**Figure 4:**
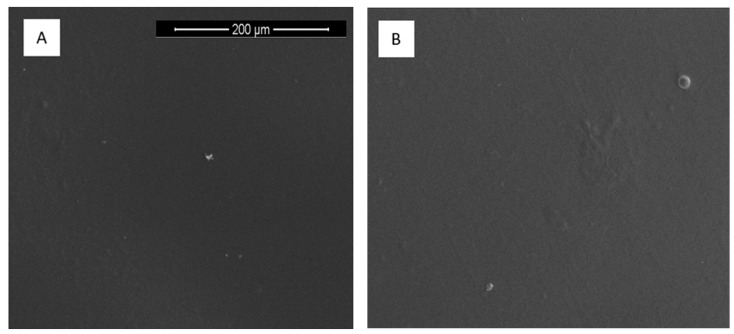

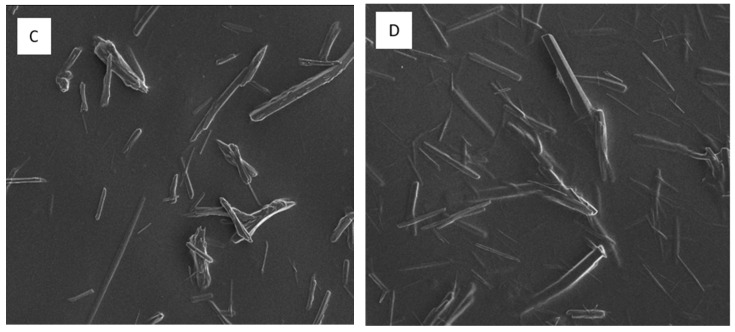
SEM images of thin films based on collagen with (**A**) 1% FA; (**B**) 2% FA; (**C**) 5% FA; and (**D**) 10% FA (magnification: 500×).

Scanning electron microscope was used to observe the morphology of thin films obtained by solvent evaporation (acetic acid) from collagen/ferulic acid complexes. The morphology of collagen-based films with ferulic acid is different depending on the FA content (Figure 4). Low FA addition (1 and 2%) allows one to obtain thin films without any irregularities on the prepared film surface. Higher FA addition such as 5% and 10% results in the precipitation of longitudinal structures on its surface. The increase of ferulic acid addition causes the increase of material heterogeneity. 

### 3.3. Mechanical Properties

**Figure 5:**
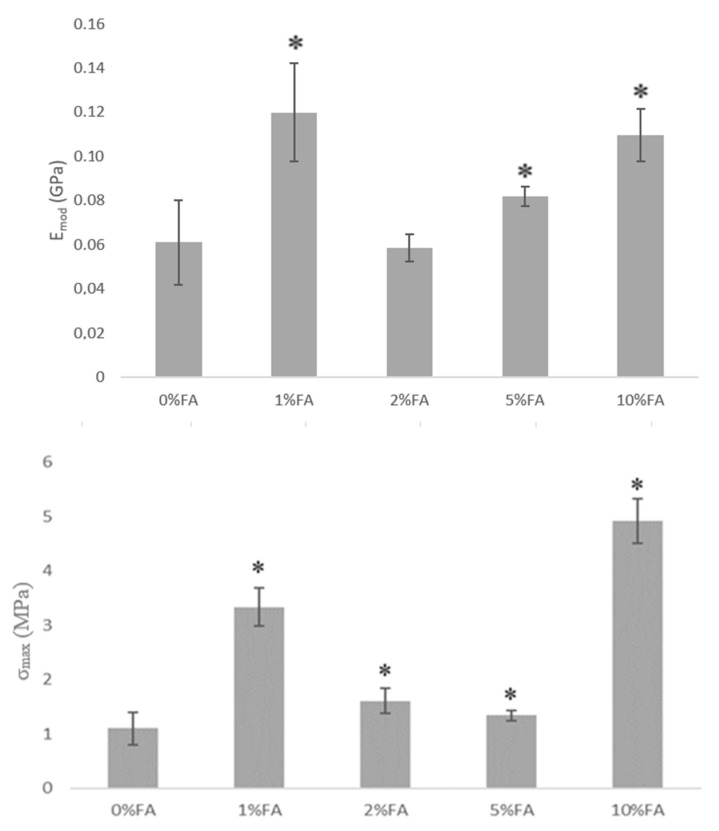

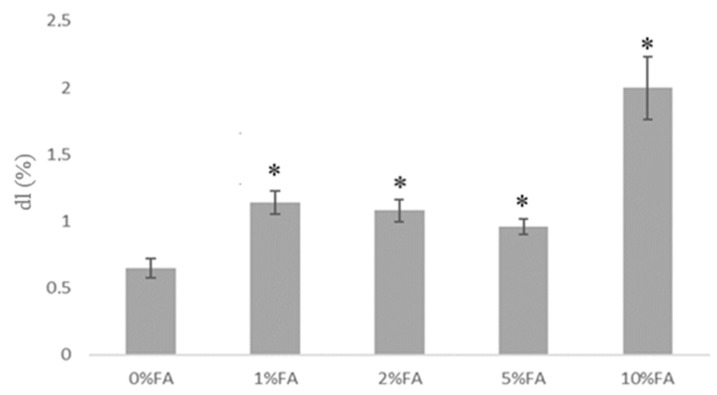
The mechanical parameters of collagen thin films with ferulic acid addition: (E_mod_—Young Modulus; σ_max_: maximum tensile strength; dl: elongation at break; n = 5; mean ± SD, *: significantly different between the groups—*p* < 0.05 vs. 0% FA; SD—standard deviation).

Figure 5 presents mechanical parameters such as Young Modulus, maximum tensile strength, and elongation at break for collagen films with ferulic acid addition. 

As it can be seen in Figure 5, Young modulus was similar for films with 1% ferulic acid addition and 10%. For low FA addition films have higher stiffness than with 2%, 5%, or 10%. Such alterations of mechanical properties are related to the ferulic acid properties, as they act as a cross-linker for collagen. The maximum tensile strength and elongation at break were significantly higher for films with 10% FA. Low ferulic acid content is sufficient for collagen cross-linking; however, higher ferulic acid amount is precipitated on the film surface, which suggests that cross-linking process is less effective at the molecular level. Nevertheless, complex of collagen with 10% ferulic acid allows one to prepare films with improved elastic properties. 

### 3.4. Steady Shear Measurements

**Figure 6:**
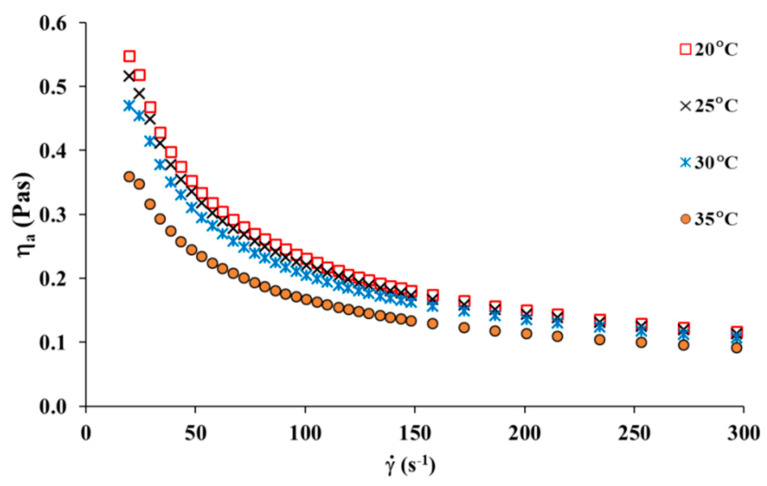
Viscosity curves of the collagen solution in temperature range 20 °C up to 35 °C.

Figure 6 presents the viscosity curves as a function of shear rate for the collagen solution at temperatures ranging from 20 to 35 °C. As it can be observed for collagen solution, a shear thinning behaviour (pseudoplastic nature) was observed, which means the decrease of apparent viscosity values according to the increase of shear rate. It is in accordance with previously reported data [26,27,28]. The shear thinning behaviour is related to the orientation of the collagen chains along the stream line of the flow and to the disentanglement of chains with the increasing shear force. The more chains are oriented, the lower the apparent viscosity of polymer solution is observed. Figure 6 also shows the viscosity curves versus shear rate of collagen solution at different temperatures. It can be noticed that temperature showed weak effect on the apparent viscosity, which just decreased slightly with increasing temperature, especially for a higher shear rate ($\dot{\gamma }$ > 150 s^−1^). 

**Figure 7:**
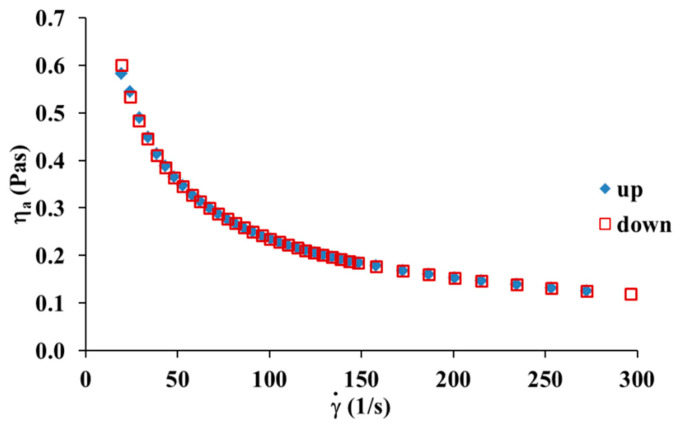
Viscosity curves hysteresis for 5 mg/mL collagen solution at 20 °C.

Measuring the viscosity curves in the experiment carried out up and down is the way to evaluate the thixotropic behaviour [26]. Figure 7 shows the viscosity curve hysteresis of a collagen solution obtained with the increasing and decreasing shear rates at 20 °C. It can be seen that the hysteresis loop did not occur for the collagen solution. Thus, this test confirms that the collagen solution used in this study is a rheologically stable fluid in which the rheological properties do not change over time.

**Figure 8:**
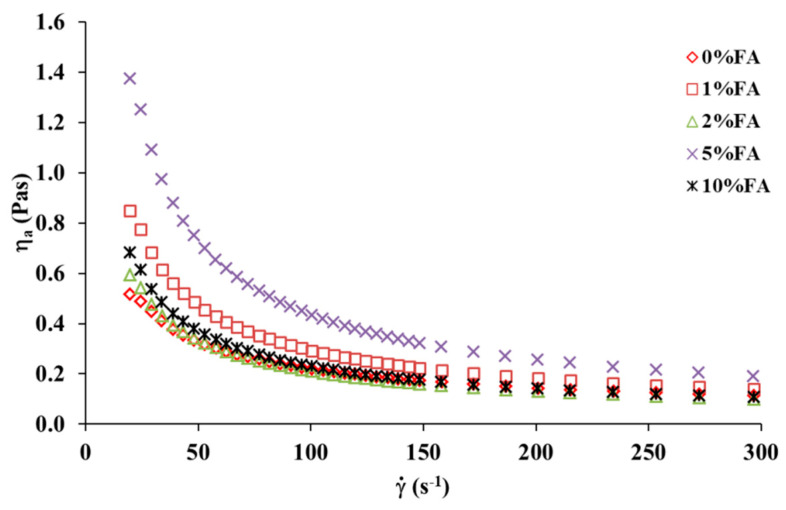
Viscosity curves of the collagen solutions with different additions of ferulic acid (FA) at 25 °C.

Figure 8 presents the viscosity curves of the collagen solutions with different additions of ferulic acid. As it can be observed, the collagen solution with 5% addition of FA has the highest apparent viscosity. For the collagen solutions with other additions of FA (1%, 2%, and 10%), no significant difference in the apparent viscosity was observed depending on different additions of FA to the polymer solution.

The shear thinning behaviour of collagen solutions with different additions of ferulic acid were successfully described by Ostwald de Waele model (Equation (1)) that has provided a good adjustment of the experimental data. The rheological parameters obtained by Ostwald de Waele model are summarized in Table 1. The value of *k* was proportional to the addition of FA and that of *n* showed an inverse dependence on FA addition with values less than 1, indicating that shear-thinning behaviour becomes more obvious with increasing the addition of FA. Moreover, the rheological parameters are practically constant with increasing temperatures. 

The activation energy of viscous flow of the collagen solutions with different additions of FA is tabulated in Table 2. 

After the addition of 1% FA to the collagen solution, we observed a slight decrease in the activation energy values for the collagen solutions; however, after addition of 2% and 10% of FA, an increase of activation energy was observed. The lowest *E*_a_ values were found for the collagen solution with 5% addition of FA. 

The observed changes in rheological properties (*η_a_* and *E_a_*) are associated with different types of interactions after different additions of FA. The rheological results showed that cross-linking reaction occurring in the collagen solution with 2% and 10% FA addition and that they are responsible for the *E_a_* increase upon FA addition, while in the collagen solution with 5% addition of FA attractive forces via hydrogen bonds play a leading role. Therefore, the different additions of ferulic acid (FA) are beneficial to the formation of new intermolecular forces, the network structure of collagen solutions, and thin films, which leads to an improvement of the properties of these materials. For modification of collagen properties in solution with 5% FA addition, the best results are obtained. In the case of thin films, the collagen film with 10% FA addition are the best results.

## 4. Discussion

Ferulic acid added to collagen modifies its secondary structure, as it probably acts as cross-linker. Ferulic acid interacts with hydrophilic groups of collagen, and as a result strong hydrogen bonds are formed. However, high ferulic acid addition (10%) did not allow for effective cross-linking process, as it was precipitated after the solvent evaporation. 

The goal of carrying out the cross-linking process of biopolymers is to improve their properties. The addition of ferulic acid to collagen results in the increase of mechanical parameters. The elastic thin films are beneficial for biomedical application, where their flexibility allows them to thoroughly cover defected skin. 

The pseudoplastic nature of collagen solution has been proved by us and is related to previous studies [26,27,28]. Polymeric chain is loose, as it is not cross-linked and stiffened by the intermolecular interactions. The measurement of solutions viscosity did not show the thixotropic behavior. However, it has been shown that collagen solution is a rheologically stable fluid as its properties do not change over time. The addition of ferulic acid to collagen solution results in the complex formation. The behavior of collagen/ferulic acid solution describes Ostwald de Waele model. It influences the solution properties that depend on the ferulic acid amount. The rheology parameters of solutions with different amount of ferulic acid were constant with increasing temperatures. Similar results were obtained by Szwajgier et al. [31], where the ferulic acid addition to the whey protein solution resulted in the improvement of rheological properties. The lower viscous flow activation energy caused a smaller energetic barrier for segmented motions in the confined space. We did not observe any trend of activation energy values with the change of ferulic acid content [32]. However, higher FA concentration added to the collagen (2 and 10%) caused the increase of activation energy, whereas 5% addition caused slight decrease of activation energy. Thereby, higher ferulic acid content caused the increase of energetic barrier as a complexation of collagen occurred. It has been reported by Prasad et al. [33] that hydrogen bonds formed between polymers and phenolic acids are unusually strong. As solutions of collagen and ferulic acid are prepared with low pH, hydrogen bonds can form rather than ionic interactions. However, covalent bonds formation cannot be completely excluded as some scientists put it in their research papers. We would like to emphasize that strong hydrogen bonds are formed between collagen and ferulic acid [34,35]; nevertheless, we did not notice any strong covalent bonds formation between them when dissolved in acetic acid using viscometry techniques. It is probable that pH of solution may indicate the change of interaction nature. As collagen is soluble in acidic pH, it is not easy to find a way to compare interaction between collagen and ferulic acid in pH higher than 7. 

Taking into account all the results obtained in our study, we can say that 1% of ferulic acid addition improves mechanical properties of collagen films. This small amount of ferulic acid is sufficient for improving collagen properties for use in humans and patients, for example, in wound healings and tissue engineering.

## 5. Conclusions

Ferulic acid has been tested as a collagen cross-linker in solution and in thin films. As a result of strong hydrogen bonds formation between collagen and FA, the complex structure was formed. Thin films prepared from collagen with 1% and 2% ferulic acid addition were homogeneous, which indicates effective interactions via hydrogen bonds and the cross-linking process between collagen and FA. Films with 5% and 10% ferulic acid addition had a heterogeneous surface, as ferulic acid did not bind effectively. Prepared thin films were elastic and stable, as cross-linking process improves material’s properties. Collagen solution showed rheological properties characteristic for biopolymers without any significant change after ferulic acid addition, but for collagen solution with 5% addition of FA the apparent viscosity was the highest. It may be assumed that ferulic acid may be proposed as a chemical agent with antioxidant activity for modification of collagen properties in solution and in thin films. 

## Figures and Tables

**Table 1:** The rheological parameters from Ostwald de Waele model for collagen solution as function of temperature and additions of FA.

T (°C)	*n*	*k* (Pa s)^n^	R^2^
0% FA			
20	0.41	3.15	0.998
30	0.43	2.79	0.998
1% FA			
20	0.33	5.98	0.999
30	0.35	5.28	0.999
2% FA			
20	0.33	4.01	0.999
30	0.35	3.59	0.999
5% FA			
20	0.26	13.2	0.998
30	0.27	11.2	0.998
10% FA			
20	0.31	5.47	1
30	0.34	4.15	0.999

*n* and *k* are rheological parameters.

**Table 2:** *E*_a_ value for the collagen solutions.

c_FA_ (%)	*E*_a_ (kJ/mol)					
	43.3 s^−1^	R^2^	91.0 s^−1^	R^2^	158 s^−1^	R^2^
0	10.75	0.997	9.29	0.986	7.97	0.997
1	10.72	0.997	8.86	0.99	7.75	0.995
2	12.31	1	10.26	0.999	8.87	0.998
5	8.81	0.981	7.15	0.989	7.08	0.989
10	11.43	0.998	8.82	0.995	7.55	0.993

**c_FA_**—concentration of ferulic acid.
